# A pancreatic zone at higher risk of fistula after enucleation

**DOI:** 10.1186/s12957-018-1476-5

**Published:** 2018-08-29

**Authors:** Pauline Duconseil, Ugo Marchese, Jacques Ewald, Marc Giovannini, Djamel Mokart, Jean-Robert Delpero, Olivier Turrini

**Affiliations:** 1 Department of Surgery, Institut Paoli-Calmettes, Marseille, France; 2 Department of Endoscopy, Institut Paoli-Calmettes, Marseille, France; 3 Department of Intensive Care, Institut Paoli-Calmettes, Marseille, France; 4 Department of Surgery, Aix-Marseille University, Institut Paoli-Calmettes, CNRS, Inserm, CRCM, Marseille, France

**Keywords:** Enucleation, Pancreatic fistula, Magnetic resonance imaging

## Abstract

**Background:**

To determine predictive factors of postoperative pancreatic fistula (POPF) in patients undergoing enucleation (EN).

**Methods:**

From 2005 to 2017, 47 patients underwent EN and had magnetic resonance imaging available for precise analysis of tumor location. Three pancreatic zones were delimited by the right side of the portal vein and the main pancreatic head duct (zone #3 comprising the lower head parenchyma and the uncinate process).

**Results:**

The mortality and morbidity rates were 0% and 62%, respectively. POPF occurred in 23 patients (49%) and was graded as B or C (severe) in 15 patients (32%). Four patients (8.5%) developed a postoperative hemorrhage, and 5 patients (11%) needed a reintervention. In univariate and multivariate analyses, the pancreatic zone was the unique predictive factor of overall (*P* = .048) or severe POPF (*P* = .05). We did not observe any difference in postoperative courses when comparing the EN achieved in zones #1 and #2. We noted a longer operative duration (*P* = .016), higher overall (*P* = .017) and severe POPF (*P* = .01) rates, and longer hospital stays (*P* = .04) when comparing the EN achieved in zone #3 versus that in zones #1 and #2. Patients who underwent EN in zone #3 had a relative risk of developing a severe POPF of 3.22 compared with patients who underwent EN in the two other pancreatic zones.

**Conclusion:**

Our study identifies the lower head parenchyma and the uncinate process as a high-risk zone of severe POPF after EN. Patients with planned EN in this zone could be selected and benefit from preoperative and/or intraoperative techniques to reduce the severe POPF rate.

**Electronic supplementary material:**

The online version of this article (10.1186/s12957-018-1476-5) contains supplementary material, which is available to authorized users.

## Background

Parenchyma-sparing pancreatectomies were proposed as an alternative to standard pancreatectomy for noninvasive tumors to avoid pancreatic endocrine and exocrine insufficiencies [1–4]. In this manner, enucleation (EN) was first performed in the 1960s [5]; currently, neuroendocrine tumors (particularly insulinoma) and branch-duct intraductal papillary mucinous neoplasms (IPMNs) are the more frequent tumors resected by EN [6–15]. As EN induces parenchyma incision and, occasionally, deep pancreas opening, it exposes patients to postoperative pancreatic fistula (POPF) by unknown main pancreatic duct injury or weakening, especially if thermo-coagulation has been used too closely. Consequently, several reports have shown that EN leads to the same or a higher rate of POPF than does standard pancreatectomy, but with a “toward zero” mortality [4, 7, 10, 12, 15–17]. Predictive factors of POPF after EN have already been reported, such as age [6], body mass index [10], distance from the main pancreatic duct (≤ 2 mm) [17], cystic morphology [6], history of acute pancreatitis [6], and New York Heart Association class [14] (Table 1). These factors are not a contraindication for EN but could help pancreatic surgeons inform patients about possible prolonged postoperative courses, counterbalanced by the very low risk of developing diabetes mellitus and/or steatorrhea [1–4]. The pancreatic location of the EN (i.e., the head/uncinate process versus body/tail) seemed to be a relevant factor of POPF [9, 10, 13] (Table 1), but the head and uncinate process are usually considered a single location. Indeed, the uncinate process is defined by a portion of the head of the pancreas that hooks around posterior to the superior mesenteric vessels but is actually difficult to delimit from the proper head parenchyma on preoperative imaging and during surgery.

**Table 1:** Reported risk factors of POPF after enucleation

	*n*	Head and uncinate together	POPF	Severe POPF	Risk factors
Turrini et al. [16]	7	Yes	43%	14%	–
Brient et al. [17]	52	Yes	27%	14%	Distance to MPD ≤ 2 mm
Jilesen et al. [10]	60	Yes	–	31%	Head/uncinate BMI
Kaiser et al. [8]	74	Yes	46%	27%	–
Faitot et al. [13]	126	Yes	57%	41%	Head/uncinate
Song et al. [9]	65	Separated	20%	9%	Head/uncinate
Strobel et al. [11]	166	Yes	41%	21%	Cystic morphology
Wang et al. [6]	142	Yes	53%	16%	Age Acute pancreatitis Cystic morphology
Zhang et al. [14]	119	Yes	40%	28%	NYHA class
Present series	47	Separated	49%	32%	Lower head + uncinate

*BMI* body mass index, *POPF* postoperative pancreatic fistula, *NYHA* New York Heart Association

The present study, based on a precise tumor location on pancreatic magnetic resonance imaging (MRI), seeks to determine predictive factors of POPF in patients who underwent EN.

## Methods

### Initial population

From January 1, 2005, to December 31, 2017, 95 patients were eligible for EN at Institut Paoli-Calmettes (Marseille, France). All patient data were entered into a clinical database (CHIRPAN database: N°Sy50955016U) approved by both the Institut Paoli Calmettes Institutional Review/Ethical and the CNIL (“Commission Nationale de l’Informatique et des Libertés” the French national board for databases) boards. Eligibility criteria for EN were (a) neuroendocrine or cystic tumor (side branch IPMN without worrying features, mucinous cystadenoma), (b) absence of main pancreatic duct dilatation, (c) absence of mural nodule or thickness of cyst wall, and (c) ability to preserve the main pancreatic duct.

### Initial staging and final selected population (Fig. 1)

**Fig. 1:**
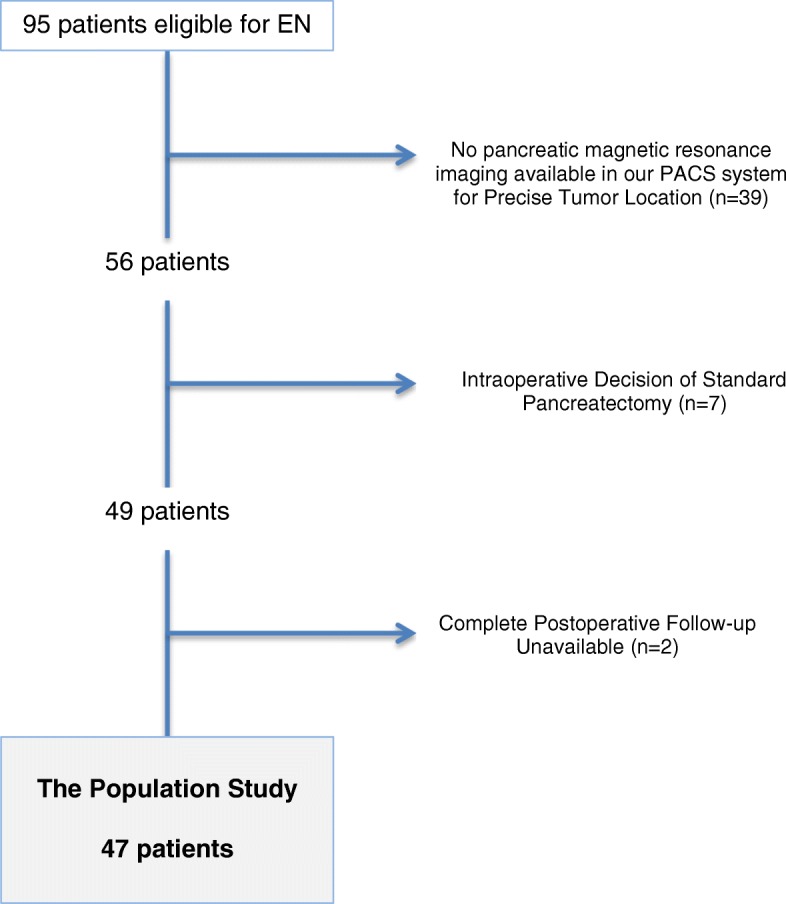
Patient selection for the present study

**Fig. 2:**
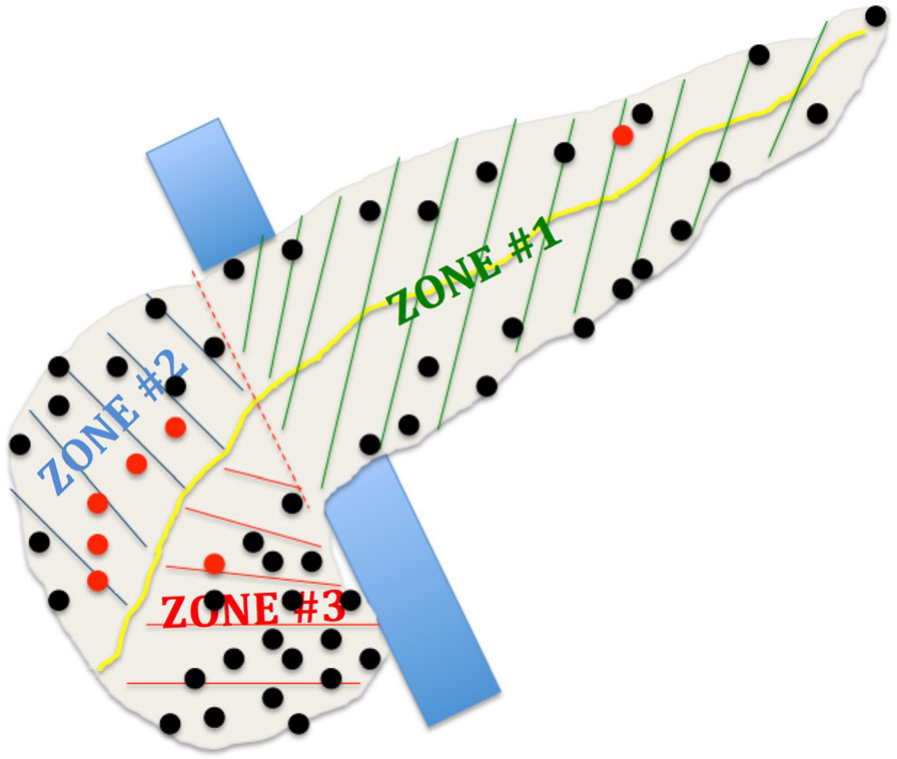
Pancreatic zones of enucleation and location of tumors (center) according to pancreatic MRI

All patients were staged by physical examination, endoscopic ultrasound, and thin-section contrast-enhanced helical dual-phase scanning. As we supported that MRI was the most relevant imaging exam to assess pancreatic tumors [18], only patients who had a recent preoperative (within the month before surgery) MRI available on our picture archiving and communication system were included in the present study (*n* = 56) (Fig. 1). Consequently, for each EN, we could precisely determine the tumor location based on (a) the center of the tumor, for a spherical tumor, and (b) the crossing point between length and width, for a non-spherical tumor, as well as the tumor size (mm), and the distance to the main pancreatic duct (mm). Three pancreatic zones were arbitrarily delimited by the right side of the portal vein and the main pancreatic head duct; consequently, the pancreatic head was divided into two easily identifiable zones: zone #2, corresponding to the upper head parenchyma, and zone #3, comprising the lower head parenchyma and the uncinate process (Fig. 2). Zone #1 grouped the body and tail of the pancreas from the right side of the mesenteric vessels to the left parenchyma. Patients whose postoperative courses were not precisely known (within 90 postoperative days) were also excluded (*n* = 2) (Fig. 1).

### Surgery

EN was achieved through laparotomy or laparoscopy according to surgeon preference and tumor location. Direct intraoperative ultrasound exploration was routinely performed to identify the main pancreatic duct and its distance to the tumor. Seven patients (5 in zone #2, 1 in zone #1, and 1 in zone #3) did not undergo EN because the tumor directly contacted the main pancreatic duct on intraoperative ultrasound; consequently, these patients with tumor located in zones #2 and #3 underwent pancreaticoduodenectomy and the patient with tumor located in zone #1 underwent distal pancreatectomy. Finally, 47 patients underwent EN and formed our population for the present study (Fig. 1); their tumor locations are presented in Fig. 2. The operative technique has already been described for pancreatic head IPMNs [16], and the global technical approach was not different for the body/tail or other cystic/solid tumors; coagulation was avoided to the greatest extent possible to avoid exposing the main pancreatic duct to thermal injury (Additional file 1: Figure S1). In cases of EN in zones #2 and #3, an intraoperative cholangiography with retrograde contrast back filling of the pancreatic duct was achieved to assess the integrity of both the biliary and pancreatic ducts. If the tumor was located in the “posterior” zone #3 (i.e., on the posterior face of the head of the pancreas), a larger Kocher maneuver was achieved to optimally expose the EN area. A non-aspirating drain was left in contact with the EN area, and the drain amylase level was measured on postoperative days 1 and 3. No patient had had a preventive preoperative stent inserted in the main pancreatic duct. Octreotide was not routinely used in prevention or with curative intent in the case of a POPF diagnosis.

### End-points studied

The variables evaluated included age, sex, body mass index, location of the tumor (i.e., zone #1, #2, or #3), laparoscopic or open approaches, operative duration (minutes), POPF according to ISGPF grading [19] (grade A fistula was defined by a drain amylase level threefold higher than the serum amylase level; grades B and C defined severe POPF), and the overall morbidity [20] rate, including postoperative bleeding, reoperation, and perioperative red cell transfusion rates; endoscopic or radiologic drainage of a deep collection was noted, as was interventional embolization. The length of hospital stay (days) and the readmission rate were also recorded. The need of a standard pancreatectomy (salvage pancreatectomy) due to unresolved POPF was noted. Tumor morphology (i.e., cystic or solid (mixed tumors were considered as cystic type)) and exact histologic denomination (i.e., neuroendocrine tumor, IPMN, and others) were the recorded histological criteria.

### Statistical analyses

The categorical factors were compared using Fisher’s exact test, and the continuous variables were compared using Student’s *t* test. Significance was set after a two-sided *P* ≤ .05. Prognostic factors with a *P* < .1 in univariate analysis or that were known to be relevant to predicting POPF were entered into a multivariable regression model to determine the independent predictors. Data analyses were performed using GraphPad Prism software, version 5.0d (GraphPad Software Inc., La Jolla, CA, USA) and SAS statistical software version 9.1 (SAS Institute, Inc., Cary, NC, USA).

## Results

### Patient characteristics (Table 2)

Patients were mainly women (74%), with a median age of 55 years (range, 19–78) and a median BMI of 22.7 (range, 18–35). Tumors were located in zone #1 in 20 patients (43%), in zone #2 in 9 patients (19%), and in zone #3 in 18 patients (38%) (Fig. 2); the most common morphology was cystic (53%), and the median size was 20 mm (range, 4–61). Twenty-one patients (45%) had a tumor distance from the main pancreatic duct ≤ 2 mm. Among patients who underwent EN in zone #3, 6 (33%) had an uncinectomy.

**Table 2:** Patients and tumor characteristics. Intraoperative data and postoperative courses

	*n* or median	% or range
Male gender	12	26%
Age (years old)	55	19–78
BMI (kg/m^2^)	22.7	18–35.1
Pancreatic zones		
1	20	43%
2	9	19%
3	18	38%
Distance to main duct (mm)		
≤ 2 mm	21	45%
> 2 mm	26	55%
Indication		
Neuroendocrine tumors	20	43%
Branch-duct IPMN	17	36%
Others	18	21%
Tumor morphology		
Cystic	25	53%
Solid	22	47%
Tumor size (mm)	20	4–61
Operative duration (min)	180	90–400
POPF		
Grade A	8	17%
Grades B and C	15	33%
Total	23	50%
Delay surgery—POPF	7	4–26
Hemorrhage	4	8.5%
Reintervention	5	11%
Dindo morbidity		
Grades 3–4	16	34%
Overall	29	62%
Perioperative transfusion	4	8.5%
Length of hospital stay (days)	15	6–90
Readmission	3	6.4%

*BMI* body mass index, *IPMN* intraductal papillary mucinous neoplasm, *POPF* postoperative pancreatic fistula)

### Surgery and postoperative course (Table 2)

The median operative duration was 180 min (range, 90–400). We detected one (2.1%) main pancreatic duct injury (in zone #3) according to intraoperative cholangiography: main pancreatic duct was electively closed with interrupted stich (Prolen 7/0) controlled by another cholangiography; a large drainage was positioned close to the suture. The mortality and overall morbidity rates were 0% and 62%, respectively. Dindo grade 3–4 complications were diagnosed in 16 patients (34%). POPF occurred in 23 patients (49%) and was severe in 15 patients (32%). The median delay between EN and POPF diagnosis was 7 days (range, 4–26). Four patients (8.5%) developed a postoperative hemorrhage in the EN zone, who all required red blood cell transfusion (range, 2–6 units). Five patients (11%) needed a reintervention for hemorrhage (*n* = 4) or duodenal fistula (*n* = 1) (cf infra). No patient required a salvage pancreatectomy. The length of hospital stay was 19 days (range, 6–90). Three patients (6.4%) needed a readmission for medical complications (urinary infection (*n* = 2), benign pulmonary embolism (*n* = 1)).

### Pancreatic fistula and risk factors (Table 3a and b)

All patients (*n* = 8) with grade A POPF (of whom three patients with main pancreatic duct direct leakage (all after EN in zone #3) including the patient with main pancreatic duct injury detected intraoperatively) were managed with continuous normal alimentation and progressive drain withdrawal; the median POPF resolution delay (i.e., from POPF diagnosis) was 9 days (range, 4–16). Among the 15 patients with severe POPF, 12 (80%) required an endoscopic or radiologic drainage of a deep collection, 4 patients (27%) required a reintervention for hemorrhage, and one patient (6.7%) developed a duodenal fistula treated by conservative surgery (i.e., direct suture and drainage with progressive fistula spontaneous closure). Two patients who developed a hemorrhage needed a reintervention after an attempt at interventional embolization because the responsible artery could not be reached; another two patients underwent direct reintervention due to bleeding of the gastric wall after a transgastric drainage of a deep collection (Additional file 2: Figure S2a–e).

**Table 3:** Univariate and multivariate analysis of risk factor for (a) all grade of postoperative pancreatic fistula and (b) grades B to C of postoperative pancreatic fistula

a)	POPF (*n* = 23)%	No POPF (*n* = 24)%	Univariate *P* value	Odd ratio [95% CI]	Multivariate *P* value
Gender				–	–
Male	8 (35)	4 (17)	.19		
Female	15 (65)	20 (83)			
Age (years old)	52	56	.39	–	–
BMI (kg/m^2^)	24.6	22 .7	.17	–	–
Pancreatic zones			.03	2.03 **[**1.01–4.07**]**	.048
1	8 (35)	12 (50)			
2	2 (9)	7 (29)			
3	13 (56)	5 (21)			
Distance to main duct (mm)				–	–
≤ 2 mm	10 (44)	11 (46)	1		
> 2 mm	13 (46)	13 (54)			
Tumor morphology			1	–	–
Cystic	13 (47)	12 (50)			
Solid	10 (44)	12 (50)			
Tumor size (mm)	24	22	.55	–	–
Laparoscopic approach	5 (22)	6 (25)	1	–	–
Operative duration (min)	205	180	.26	–	–
b)	POPF B/C (*n* = 15)%	No POPF B/C (*n* = 32)%	Univariate *P* value	Odd ratio [95% CI]	Multivariate *P* value
Gender			.16	–	–
Male	6 (40)	6 (19)			
Female	9 (60)	26 (81)			
Age (years old)	54	53	.85	–	–
BMI (kg/m^2^)	24.6	23.1	.32	–	–
Pancreatic zones			.02	2.06 **[**1.01–4.3**]**	.05
1	3 (20)	17 (53)			
2	2 (13)	7 (22)			
3	10 (67)	8 (25)			
Distance to main duct (mm)			.15		
≤ 2 mm	9 (60)	12 (38)		–	–
> 2 mm	6 (40)	20 (62)			
Tumor morphology			.99		
Cystic	8 (53)	17 (53)		–	–
Solid	7 (47)	15 (47)			
Tumor size (mm)	22	22	.98	–	–
Laparoscopic approach	2 (13)	9 (28)	.27	–	–
Operative duration (min)	200	190	.53	–	–

*BMI* body mass index, *POPF* postoperative pancreatic fistula, *CI* confidence interval

In the univariate and multivariate analyses, the pancreatic zone was the unique predictive factor of overall (*P* = .048) or severe (*P* = .05) POPF. Distance to the main pancreatic duct and cystic morphology was not identified as a relevant factor for predicting overall or severe POPF.

### Pancreatic zones of enucleation (Table 4)

**Fig. 3:**
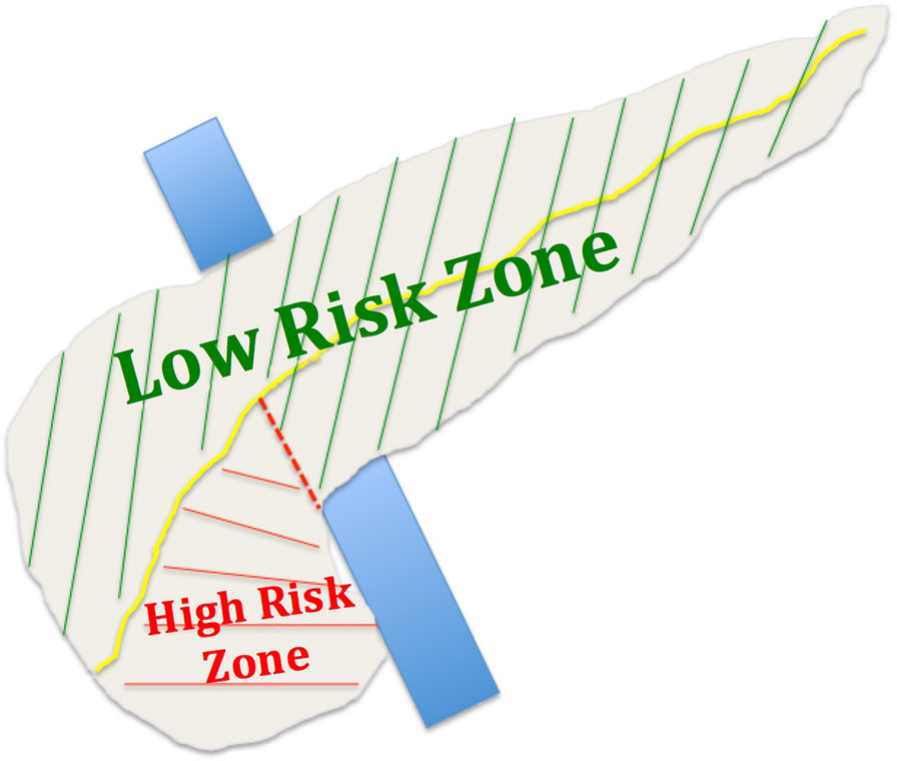
Pancreatic zones to predict overall and severe POPF according to pancreatic MRI

The patient and tumor characteristics were not different among the three groups defined by the three zones. Laparoscopic approach was more often used for EN in zone #1 + 2 when compared with zone #3 (*P* = .03). We did not observe any difference in postoperative courses when comparing the EN achieved in zones #1 and #2. We noted a longer operative duration (*P* = .016), higher overall (*P* = .017) and severe (*P* = .01) POPF rates, and longer hospital stay (*P* = .04) when comparing the EN achieved in zone #3 versus that in zone #1 + 2. Patients who underwent EN in zone #3 had a relative risk [95% confidence interval] of developing a severe POPF of 3.22 [1.31–7.91] compared with patients who underwent EN in the two other pancreatic zones (Fig. 3).

**Table 4:** Patients and tumor characteristics. Intraoperative data and postoperative courses according to the pancreatic zone classification risk of POPF

	Zone #1	Zone #2	*P* value zone#1 vs zone #2	Zone #3	*P* value zone#1 + 2 vs zone #3
Tumor located in the corresponding zone but patient underwent standard pancreatectomy according to intraoperative decision (%)*	1 (5)	5 (36)	.027	1 (5)	ns
*n* (%)	20 (43)	9 (19)	–	18 (38)	–
Male gender (%)	3 (15)	1 (11)	ns	2 (11)	ns
Age (years old)	53	52	ns	56	ns
BMI (kg/m^2^)	22.8	21.5	ns	25.4	ns
Distance to Wirsung (mm)					
< 2 mm (%)	7 (35)	4 (44)	ns	10 (56)	ns
> 2 mm (%)	13 (65)	5 (56)	ns	8 (44)	ns
Tumor morphology					
Cystic (%)	10 (50)	5 (56)	ns	10 (56)	ns
Solid (%)	10 (50)	4 (44)	ns	8 (44)	ns
Tumor size (mm)	22	25	ns	22	ns
Laparoscopic approach	8 (40)	2 (22)	ns	1 (6)	.03
Operative duration (min)	170	180	ns	220	.016
POPF					
Grade A (%)	5 (25)	0	ns	3 (17)	ns
Grades B and C (%)	3 (15)	2 (22)	ns	10 (56)	.01
Total (%)	8 (40)	2 (22)	ns	13 (72)	.017
Hemorrhage (%)	0	1 (11)	ns	3 (17)	ns
Reintervention (%)	0	1 (11)	ns	4 (22)	ns
DINDO morbidity					
Grades 3–4 (%)	3 (15)	5 (56)	ns	8 (44)	ns
Overall (%)	8 (40)	5 (56)	ns	16 (89)	ns
Perioperative transfusion (%)	0	0	ns	4 (22.2)	ns
Length of hospital stay (days)	13	15		23	.04
Readmission (%)	0	0	ns	3 (17)	ns

*vs* versus, *BMI* body mass index, *POPF* postoperative pancreatic fistula

*These patients are not comprised in our study population

## Discussion

Our study identified a high-risk zone (the lower head parenchyma and uncinate process) for developing a POPF after EN, with a relative risk of 3.22 compared to the rest of the cases of pancreatic parenchyma.

### POPF after EN

It is now well known that EN can lead to the same POPF rate as standard pancreatectomies (Table 1); however, EN can be considered safer in terms of its mortality rate, which might increase to 10% [21, 22] after pancreatectomies. This finding completely justifies EN as a procedure of choice in patients with benign tumors. Consequently, the patient must be informed that EN is a “mini invasive” pancreatic procedure with the advantage of preserving pancreatic parenchyma but without a reduced risk of POPF and its associated morbidity. The risk factors of overall and severe POPF have already been studied: on the one hand, several factors, such as age [6], body mass index [10], distance to the main pancreatic duct [17], history of acute pancreatitis [6], and New York Heart Association class [14], were independently identified by a single publication each (Table 1); on the other hand, cystic morphology [6, 11] and tumor location [9, 10, 13] were identified in at least two independent series (Table 1). Our study confirmed that tumor location (i.e., the corresponding EN area) was relevant to predict POPF, but we improved the precision of this result by delimiting a particular zone with a higher risk than the rest of the pancreatic parenchyma. Despite the increasing trend, we did not observe any difference in terms of overall postoperative complications, postoperative hemorrhage, reintervention, perioperative transfusion, and readmission rates between patients who underwent EN in the high-risk zone and the others; however, these factors are clearly directly related to severe POPF, and the reduced sample of our series might be the cause of the non-significance of these results.

### Pancreatic zones

As the head of the pancreas is a more difficult zone to expose and represents the intersection of the pancreatic, biliary, and digestive tracts, it was supposed to be at a higher risk of complication after EN. This has already been reported [9, 10, 13], but the uncinate process has never been studied separately from the rest of the head of the pancreas. Our pancreatic partition based on MRI was efficient for easily identifying patients at a high risk of developing a severe POPF after EN. We observed that patients who underwent EN of a tumor located in the upper part of the head (i.e., in zone #2) had a similar risk of experiencing severe POPF as did patients who underwent EN for a tumor located in the body or tail of the pancreas. We suppose that patients who underwent EN in zone #2 were carefully selected and that their tumors probably not deeply located in the head; in such cases, due to the high probability of bile duct injury, a pancreaticoduodenectomy may have been preferred preoperatively. This was confirmed by the lower number of patients (19% of our studied population) who underwent EN in zone #2 and by the higher rate of patients with a planned EN who ultimately had a standard pancreatectomy performed (36% in zone #2, 5% in zone #1, and 5% in zone #3). Thus, our study is limited by the absence of study of the tumor’s depth as a risk factor of POPF, even if it has never been highlighted in another study [13].

### Prevention of POPF in the high-risk zone

Our study should not discourage pancreatic surgeons from performing EN in patients with tumors located in the high-risk zone because we showed that (a) it was a safe procedure, despite its high morbidity rate and (b) no patients required a salvage pancreaticoduodenectomy. Indeed, our findings permitted the identification of patients who should benefit from strict follow-up and possibly from POPF-preventive procedures. Some authors have described the usefulness of preoperative stenting in patients undergoing EN to reduce main pancreatic duct leakage [23]; a nasopancreatic drain can also be inserted preoperatively to intraoperatively contrast the back-fill of the main pancreatic duct [24] and thereby identify an unknown injury. If the main pancreatic duct stenting is reported as an interesting procedure for treating a prolonged POPF [25], it is correlated with a non-negligible risk of acute pancreatitis [26, 27] and cannot be proposed as a preoperative routine procedure. Indeed, when the EN of a deep tumor is planned in the high-risk zone, a sphincterotomy followed by the insertion of a stent in the main pancreatic duct could be discussed prior to surgery. However, three arguments must be considered before routinely adopting such a policy. First, a recent randomized study did not confirm the benefit of preoperative stenting to reduce POPF [28]. However, this series was only conducted in patients who underwent distal pancreatectomy, and no data are available concerning proximal or distal EN. Second, EN in the high-risk zone also exposes the patient to other pancreatic duct injuries such as *pancreas divisum* (Type IV of the Cambridge classification; 5% of the population; risk of injury of the accessory duct) [29]; *ansa pancreatica* (Type V; 1% of the population) [29] is another duct variation in which a loop communication is made between the main and accessory duct: stenting of the sole main duct will thus not be sufficient to prevent POPF. Third, pancreatic stenting (even after stent withdrawal) in patients diagnosed with IPMN can lead to main duct modification at imaging and impair the follow-up (modifications due to main duct IPMN or stenting). We support the idea that a careful examination of both MRI and endoscopic ultrasound could help select patients who might benefit from preoperative pancreatic stenting (the patient in the second images should have been a good candidate for preoperative stenting to avoid a lateral main duct injury despite careful ligature of the communication duct). Intraoperatively, in the case of deep EN in the high-risk zone, the pancreatic surgeon could achieve en-Roux pancreaticojejunostomy [30] or teres hepatis ligament flap plasty [31], even if such procedures have not been validated in large series to reduce POPF risk. Finally, drainage of the EN zone must be optimal to avoid pancreatic juice stagnation and favor grade A instead of severe POPF. However, drainage of the “anterior” high-risk zone is difficult (“egg cup” effect, as in the patient in the second images) and often insufficient.

Highlighted by our findings, our actual policy is to achieve a sphincterotomy with main pancreatic duct stent insertion prior to EN in the high-risk zone in patients (a) whose main pancreatic duct is found to be closer than 2 mm at MRI and/or intraoperative ultrasound and/or (b) with branch-duct IPMN consequently linked with the main pancreatic duct. Evaluation of such attitude is ongoing, and we will compare the results with those of the 18 EN achieved in the high-risk zone without preoperative stenting that we have reported in the present series.

## Conclusion

Our study identifies a pancreatic zone that is at a high risk of developing a severe POPF after EN but with zero mortality. Thus, EN should be preferred to a pancreaticoduodenectomy to preserve long-term exocrine and endocrine pancreatic functions. However, we support the idea that patients who are planned for a deep EN in this zone should be selected and could benefit from some preoperative (main duct stenting) and/or intraoperative techniques (pancreaticojejunostomy and teres hepatis ligament flap plasty) to prevent severe POPF; these patients should be carefully followed up during the postoperative course, even if they have experimented with a “mini-invasive” surgery.

## Additional files

Additional file 1: **Figure S1.** Intraoperative picture showing the enucleation (yellow arrow) of an insulinoma in the “anterior” zone #3. Please note all ligatures (a) not to have any bleeding during the parenchyma opening that could disturb the identification of main pancreatic duct and (b) preferred to coagulation to avoid thermal damage of the main pancreatic duct. (JPEG 248 kb)

Additional file 2: **Figure S2.** (a) pancreatic frontal magnetic resonance imaging showing a branch-duct intrapapillary mucinous neoplasm of zone #3 (portal vein in represented in blue, pancreatic zones are delimited by interrupted red lines); the patient underwent EN in the anterior zone #3 with elective ligature of the communicant duct; (b) drainage (yellow arrow) of a deep collection in the EN zone by two double-pigtail plastic transgastric stents; (c) 24 h after drainage, the patient presented a brutal abdominal pain with hemoglobin serum level tumbling to 6 g/dL, and the CT scan showed an hematoma (red arrow) in the enucleation area without identification of the responsible artery at arteriography; consequently the patient underwent explorative laparotomy; (d) intraoperative picture showing the hematoma in zone #3 with “egg cup” effect (main part of the hematoma descending along the right mesocolon has already been removed); (e) intraoperative picture showing the ablation of the hematoma and the two double-pigtail plastic stents. Bleeding originated from the gastric wall, which was closed by an automatic stapler application after having removed the stents. (JPEG 303 kb)

## Abbreviations

EN: Enucleation
IPMN: Intraductal papillary mucinous neoplasm
MRI: Magnetic resonance imaging
POPF: Postoperative pancreatic fistula
