# Supplementary figures and images for: A pancreatic zone at higher risk of fistula after enucleation
Source: World J Surg Oncol. 2018 Aug 29;16:177. doi: 10.1186/s12957-018-1476-5 (PMC6116563; doi:10.1186/s12957-018-1476-5)

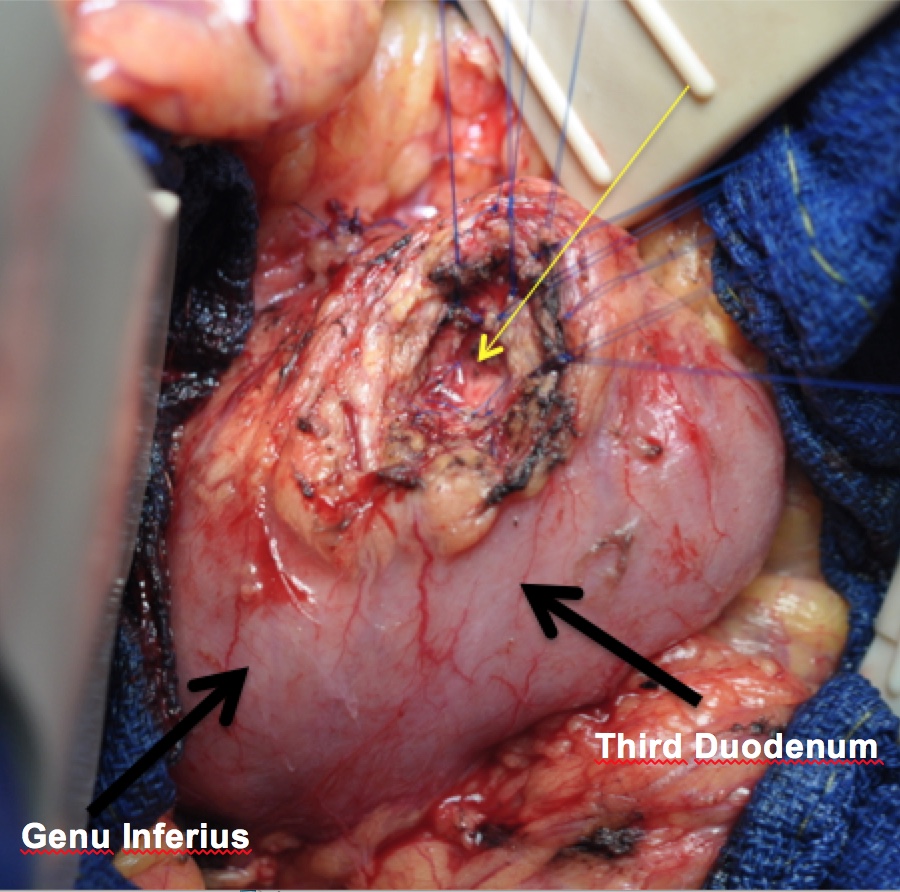

Supplement: Supplementary file 1 — Figure S1. Intraoperative picture showing the enucleation (yellow arrow) of an insulinoma in the “anterior” zone #3. Please note all ligatures (a) not to have any bleeding during the parenchyma opening that could disturb the identification of main pancreatic duct and (b) preferred to coagulation to avoid thermal damage of the main pancreatic duct. (JPEG 248 kb) [file 12957_2018_1476_MOESM1_ESM.jpeg]

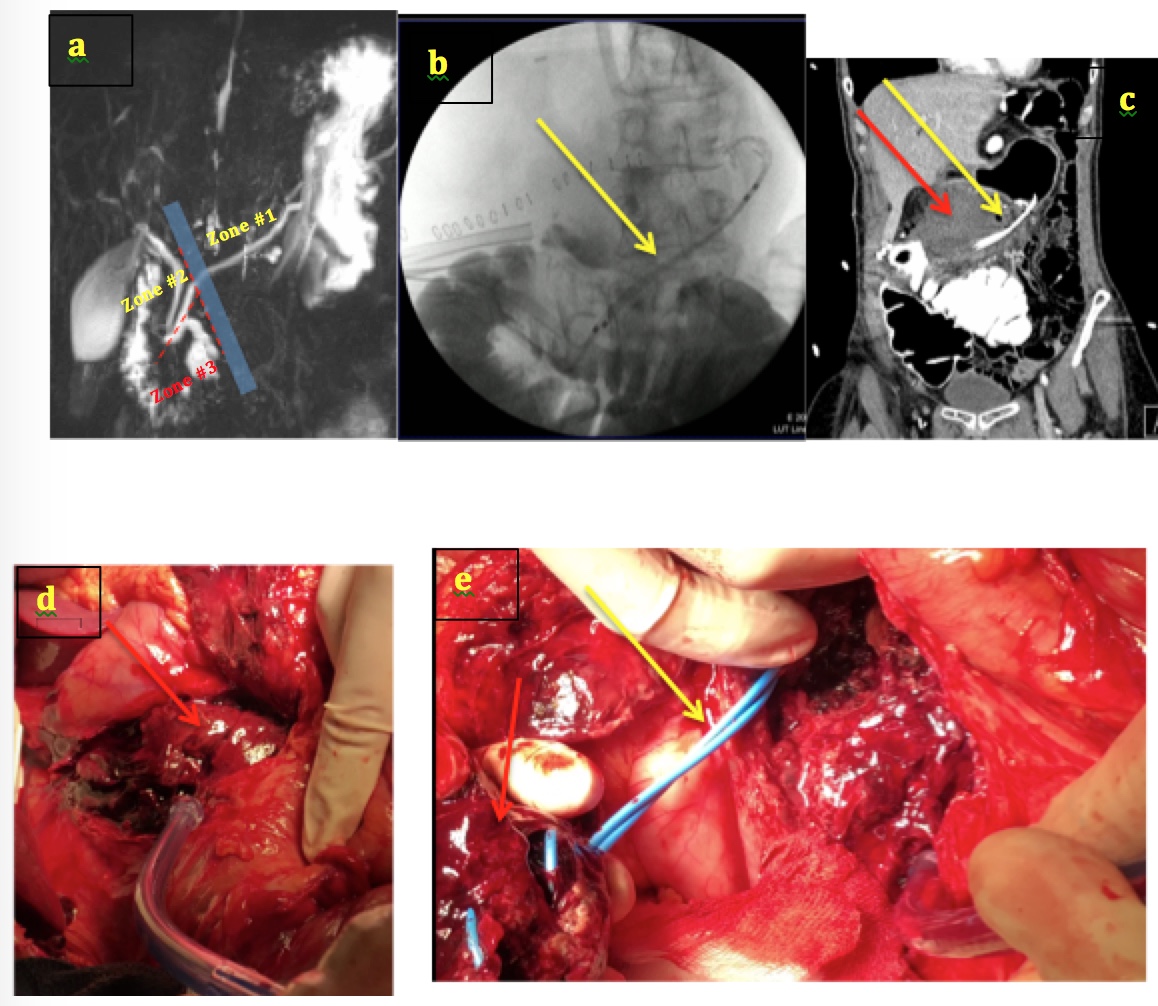

Supplement: Supplementary file 2 — Figure S2. (a) pancreatic frontal magnetic resonance imaging showing a branch-duct intrapapillary mucinous neoplasm of zone #3 (portal vein in represented in blue, pancreatic zones are delimited by interrupted red lines); the patient underwent EN in the anterior zone #3 with elective ligature of the communicant duct; (b) drainage (yellow arrow) of a deep collection in the EN zone by two double-pigtail plastic transgastric stents; (c) 24 h after drainage, the patient presented a brutal abdominal pain with hemoglobin serum level tumbling to 6 g/dL, and the CT scan showed an hematoma (red arrow) in the enucleation area without identification of the responsible artery at arteriography; consequently the patient underwent explorative laparotomy; (d) intraoperative picture showing the hematoma in zone #3 with “egg cup” effect (main part of the hematoma descending along the right mesocolon has already been removed); (e) intraoperative picture showing the ablation of the hematoma and the two double-pigtail plastic stents. Bleeding originated from the gastric wall, which was closed by an automatic stapler application after having removed the stents. (JPEG 303 kb) [file 12957_2018_1476_MOESM2_ESM.jpeg]
